# *Ficus deltoidea* Leaf Alters Oxidative Stress, Protein Homeostasis and Ubiquitin-Proteasome Pathways in Fatty Acid-Induced Cell Line

**DOI:** 10.17113/ftb.61.02.23.7802

**Published:** 2023-06

**Authors:** Noor Nazirahanie Abrahim, Norhaniza Aminudin, Puteri Shafinaz Abdul-Rahman

**Affiliations:** 1 Department of Molecular Medicine, Faculty of Medicine, Universiti Malaya, 50603 Kuala Lumpur, Malaysia; 2 Universiti Malaya Centre for Proteomics Research (UMCPR), Faculty of Medicine, Universiti Malaya, 50603 Kuala Lumpur, Malaysia; 3 Institute of Biological Sciences (IBS), Faculty of Science, Universiti Malaya, 50603 Kuala Lumpur, Malaysia

**Keywords:** *Ficus deltoidea*, steatosis, oxidative stress, ubiquitin-proteasome system, protein homeostasis

## Abstract

**Research background:**

*Ficus deltoidea* (mistletoe fig) is a shrub well known among locals in Malaysia primarily for its treatment of toothaches, colds and wounds. The aim of this study is to determine the potential of leaves, sourced from three different varieties of *F. deltoidea*, to exhibit antioxidant activity, a reduction of lipid concentration, and protein expression in steatosis-induced liver cell lines.

**Experimental approach:**

The leaves of three *F. deltoidea varieties*, namely *Ficus deltoidea* var. *angustifolia*, *Ficus deltoidea* var. *trengganuensis* and *Ficus deltoidea* var. *kunstleri*, were subjected to water extraction. The resulting crude extracts were fractionated using water and ethyl acetate. Palmitic acid was used to induce lipid accumulation (steatosis) in human liver (WRL68) cells, before all the samples were tested for their lipid-reducing activity. Several proteomic approaches were incorporated. The changes in protein expression were determined using 2-dimensional gel electrophoresis separation, whereas identification of our protein spots of interest was carried out *via* matrix-assisted laser desorption/ionization time-of-flight.

**Results and conclusions:**

*Ficus deltoidea* var. *kunstleri* alone demonstrated the ability to reduce lipids at the highest tested concentration (200 µg/mL) and was, therefore, used for subsequent experiments. Treatment with *Ficus deltoidea* var. *kunstleri* was found to restore redox status by increasing superoxide dismutase and glutathione peroxidase amounts and decreasing malondialdehyde formation. Six proteins were successfully identified; these were heat shock protein beta-1 (HSPB1), proteasome subunit alpha type 1 (PSMA1), glutathione S-transferase omega 1 (GSTO1), peroxiredoxin-1 (PRDX1), histone H2B (HIST1H2BD) and ubiquitin c-terminal hydrolase L3 (UCHL3). Through bioinformatics analysis, it was found that these proteins were significantly involved in specific pathways such as oxidative stress (PRDX1 and GSTO1), protein homeostasis (HSPB1) and degradation (UCHL3 and PSMA1).

**Novelty and scientific contribution:**

*F. deltoidea* pretreatment was shown to reduce lipid accumulation, thus improving the redox status and protein homeostasis. This suggests the role of *F. deltoidea* as a preventive mechanism in non-alcohol fatty liver disease.

## INTRODUCTION

Non-alcoholic fatty liver disease (NAFLD) is a group of liver disorders that include steatosis (excessive fat accumulation in the liver), steatohepatitis (liver inflammation), fibrosis (formation of scar tissue in the liver), cirrhosis (late stage of scarring of the liver), and the potential for progression to hepatocellular carcinoma (liver cancer). This chronic liver disease develops when there is no excessive alcohol consumption. NAFLD is a multisystem illness that affects multiple extrahepatic organs and regulatory mechanisms, according to research (*1*, *2*).

To date, there is very little effective treatment available for NAFLD besides dietary changes, regular exercise, mass loss and usage of lipid-lowering drugs. Statins, fibrates, bile acid sequestrants, niacin, ezetimibe and omega-3 fatty acids represent some of the drugs that are currently able to treat NAFLD (*3*, *4*). Despite their function as a lipid-lowering agent, some patients may develop adverse effects, especially towards statins, such as myopathy, hepatotoxicity, diabetes mellitus, and renal and neurologic decline (*5*, *6*). For this reason, there is a need for a natural source of treatment, particularly derived from plants.

In Malaysia, *F. deltoidea*, commonly known as Mas cotek is a well-known medicinal herb, especially among the Malays, and is traditionally used to treat diabetes, headaches, sore throats, and colds. Previous studies have reported its glucose-lowering effect in both animal and cell culture models (*7*, *8*). The leaves were a promising candidate for treatment of osteoarthritis by reducing the serum level of IL-1β, PGE2, PIINP and CTX-II in osteoarthritis-induced rats (*9*), while Nurdiana *et al.* (*10*) discovered the neuroprotective effects of *F. deltoidea* by improving cortical gyrification, spatial learning and memory in streptozotocin-induced diabetic rats. In addition, our previous studies demonstrated the antioxidant activities of *F. deltoidea* leaves without toxicity towards normal liver cell lines (*11*). There is no published research on the effects of *F. deltoidea* on NAFLD, and the possibility of using it for other medical conditions has not yet been adequately assessed. This work uses a cultured cell model to investigate the potential benefits of the leaves of three *F. deltoidea* varieties on NAFLD improvement, particularly on lipid accumulation.

## MATERIALS AND METHODS

### Chemicals

Ethyl acetate, iron(II) sulphate, isopropanol, dimethyl sulfoxide (DMSO), methanol and chloroform were purchased from Thermo Fisher Scientific (Leicester, UK), while low-density lipoprotein (LDL), 3-(4,5-dimethylthiazol-2-yl)-2,5-diphenyl tetrazolium bromide (MTT), bovine serum albumin (BSA) and thiobarbituric acid (TBA) were acquired from Merck (Darmstadt, Germany). Quercetin, 3-hydroxy-3-methylglutaryl coenzyme A (HMG-CoA) reductase assay kit and formaldehyde were from Sigma-Aldrich, Merck (St. Louis, MO, USA). Cell culture medium such as Dulbecco’s modified Eagle’s medium (DMEM), foetal bovine serum (FBS) and penicillin-streptomycin were from Nacalai Tesque (Tokyo, Japan). Palmitic acid and Oil Red O (ORO) were from Acros Organics (New Jersey, NJ, USA). Lastly, phosphate buffer saline (PBS) was from Oxoid (Basingstoke, UK).

### Ficus deltoidea leaf extract preparation

**Fig. S1:**
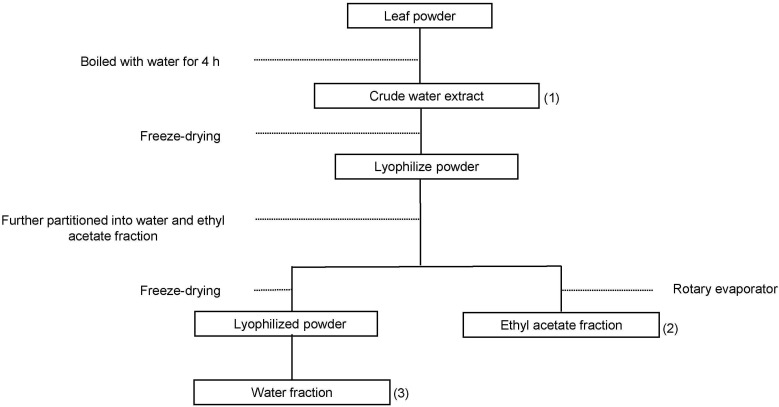
For each variety of *Ficus deltoidea* leaves, sample preparation results in crude (1), water fraction (2), and ethyl acetate fraction (3)

The leaves of three different variants of *Ficus deltoidea* species: *angustifolia* (FDA), *trengganuensis* (FDT) and *kunstleri* (FDK), were collected in Rembau, Negeri Sembilan, Malaysia. The Herbarium, Rimba Ilmu, Universiti Malaya assigned unique specimen numbers to each variation (KLU046467, KLU046469 and KLU046471). The prior publication describes how the plant extracts were obtained (*11*). Briefly, *F. deltoidea* leaves were extracted with water, followed by liquid-liquid extraction using water and ethyl acetate (Fig. S1). For each variety, subsequent tests were carried out with crude, water (WF) and ethyl acetate (EAF) fractions.

### Low-density lipoprotein oxidation assay

This assay provides a measure of the intrinsic susceptibility of the lipoproteins to be oxidised by oxidants, which in this case were Fe^2+^ ions. Using a microplate reader (Infinite M1000; Tecan, Männedorf, Switzerland), the change in the absorbance at 234 nm in 10-min intervals was monitored (Infinite M1000; Tecan) to determine the extent of LDL oxidation in relation to the generation of conjugated dienes (CD). LDL (30 g/mL) was incubated with iron(II) sulphate (10 µM) in the absence (control) or presence (0.5 g/mL) of extracts for 4 h at a constant temperature of 37 °C (*12*). The lag phase, expressed in minutes, was defined as the period when no oxidation occurred. Quercetin was used as a positive control. All analyses were performed in four replicates.

### In vitro cell-free assay and kinetic studies of 3-hydroxy-3-methylglutaryl coenzyme A reductase

This assay was performed to screen and identify the most effective crude extract and its fractions with statin-like activities. The HMG-CoA reductase inhibitory activity of the samples (5 µg/mL) was measured spectrophotometrically (Infinite M1000; Tecan) at 340 nm according to the manufacturer’s (Sigma-Aldrich, Merck) protocol. All analyses were done in triplicate. Results were expressed as a percentage of inhibition using the following formula:

Inhibition=[(*A*_c_–*A*_t_)/*A*_c_]·100 /1/

where *A*_c_ is the absorbance of the control reaction and *A*_t_ is the absorbance in the presence of the extracts.

### Cell culture and cytotoxicity of palmitic acid

Human liver cells (WRL68) were a gift of Dr Nurshamimi and Dr Shatrah from the Department of Molecular Medicine, Faculty of Medicine, Universiti Malaya. At 37.5 °C, the cells were grown in T25 flasks in DMEM supplemented with 10 % FBS and penicillin-streptomycin in 5 % CO_2_.

Cells were exposed to palmitic acid to induce fatty acid overload to simulate an *in vitro* steatosis condition. Cells (1.5·10^4^) were seeded in a 96-well plate and exposed to different concentrations of palmitic acid (10 µL), ranging from 0 to 800 µM for 24 h. The same amount of BSA added to the cells was used as a negative control. Cytotoxicity was measured after 24 h by adding 10 µL of MTT reagent (5 mg/mL) to the cells and incubating them for an additional 4 h. Isopropanol (100 µL) was added to each well in place of the medium and MTT reagent (*11*). The percentage of inhibition was calculated from the absorbance measured at 595 nm, using Eq. 1. Median inhibitory concentrations (IC_50_) were also determined. Each experiment was carried out in two independent batches, each in triplicate (*11*).

### Intracellular lipid content

Lipid absorption was quantified by using an ORO dye solution. Briefly, cells were washed with ice-cold PBS and fixed in 50 µL of 10 % formaldehyde for 1 h. After 3 changes of ice-cold PBS, 50 µL of ORO were added, incubated for another 2 h at room temperature, and rinsed with ice-cold PBS. For quantitative analysis of cellular lipids, 40 µL of DMSO were added to each well, and absorbance was monitored at 510 nm (Infinite M1000; Tecan). The examined cells were detected and quantified, and the ORO-stained cells were measured relative to the control well. This experiment was carried out in two different batches, each in triplicate. Results were expressed as absorbance at 510 nm relative to the control.

In this experiment, three different treatment groups were used: (*i*) the vehicle group (negative control, BSA), (*ii*) the palmitic acid group (positive control), and (*iii*) the treatment group, which was given the crude extract and fractions of *F. deltoidea* leaves at a final concentration of 50, 100 and 200 µg/mL for 24 h before being incubated with palmitic acid for additional 24 h. A sample that showed the most significant changes was subjected to further analysis. For the downstream experiment, treatment was carried out in a T25 flask.

### Cellular antioxidant activities

The sample that showed significant changes in lipid-reducing activity was tested using superoxide dismutase (SOD), glutathione peroxidase (GPx) and malondialdehyde (MDA) assays. Briefly, SOD and GPx activities were determined using the kits from Elabscience (Houston, TX, USA) and measured spectrophotometrically (Infinite M1000; Tecan) at 450 nm according to the manufacturer’s protocol.

For the lipid peroxidation assay, cell suspension (0.1 mL) and TBA reagent (0.5 mL) were combined, and the mixture was heated for 20 min. After allowing the mixture to cool, it was centrifuged (Universal 32R; Hettich Instruments, Tuttlingen, Germany) at 2500×*g* for 10 min at 25 °C. The absorbance of the supernatant (0.1 mL) was measured at 550 nm (Infinite M1000; Tecan) in a 96-well plate. All experiments were done in triplicate. Results were expressed as MDA in ng/100 μg of protein (*13*).

### 2-dimensional gel electrophoresis and image analysis

Changes in the cellular response of steatosis-prone WRL68 cells with the extract were investigated using a 2DE-based proteomic approach. Cell lysate (0.1 mL) was precipitated with 0.4 mL of methanol, 0.1 mL of chloroform and 0.3 mL of water. Later, 100 µg of protein was mixed with sample buffer and incubated for 1 h at room temperature. This mixture was then applied to DryStrips (*l*=13 cm, pH=3–10; GE Healthcare, Uppsala, Sweden) and rehydrated for at least 16 h. Cell lysate proteins were isoelectrically focused at 20 °C according to the following protocol: (*i*) 500 Vh, 500 V (step-and-hold), (*ii*) 1000 Vh, 1000 V (gradient), (*iii*) 16 000 Vh, 8000 V (gradient), and (*iv*) 12 000 Vh, 8000 V (step-and-hold); and second dimension separation was carried out using a homogenous pre-cast gel (*14*). Gel electrophoresis was carried out at 50 V and 40 mA/gel for 30 min and then at 500 V and 40 mA/gel for 3 h (SE600 Ruby; GE Healthcare, Princeton, NJ, USA). The gels were stained using the silver staining method (*15*).

The stained gels were then analysed using Progenesis SameSpots v. 3.0 (Nonlinear Dynamics Ltd., Newcastle, UK). Protein spots showing at least a 1.3-fold difference in average expression level were considered statistically significant (p≤0.05).

### Mass spectrometry and database search

Spots showing significant changes were identified using matrix-assisted laser desorption/ionization time-of-flight (MALDI-ToF/ToF; ABI 4800 Plus, AB Sciex, CA, USA). Proteins were identified using the MASCOT search engine (Matrix Science Ltd., London, UK). The obtained MS data were searched against *Homo sapiens* entries in the Swiss-Prot database (*16*) with the following parameters: one missed cleavage allowed, carbamidomethyl cysteine as the fixed modification, and oxidation of methionine as the variable modification. Proteins with a confidence level of ≥95 % were identified. Proteins with a score of more than 50 and a best ion score (MS/MS) of more than 43 indicated significant identity (p<0.05).

### Bioinformatics analysis

Data from the MS analysis was combined with bioinformatics data mining techniques to show the relationship between the discovered proteins and the metabolic pathways relevant to the phenomenon under study. The discovered proteins were exposed to a web-based bioinformatics software such as PANTHER (http://pantherdb.org/) and Reactome (https://reactome.org/) to understand the mechanism underlying the lipid-reducing activity of the sample (*17*, *18*).

### Statistical analysis

The Microsoft Excel statistical package (Microsoft, Redmond, WA, USA) was used to examine the results, which were presented as mean standard deviation (S.D.). The Student's *t*-test was used to determine statistical significance. In comparison to the control, differences in average values at the 95 % confidence level (p<0.05) were deemed statistically significant.

## RESULTS AND DISCUSSION

### Effect of Ficus deltoidea leaves on lipid-related assays

**Fig. 1:**
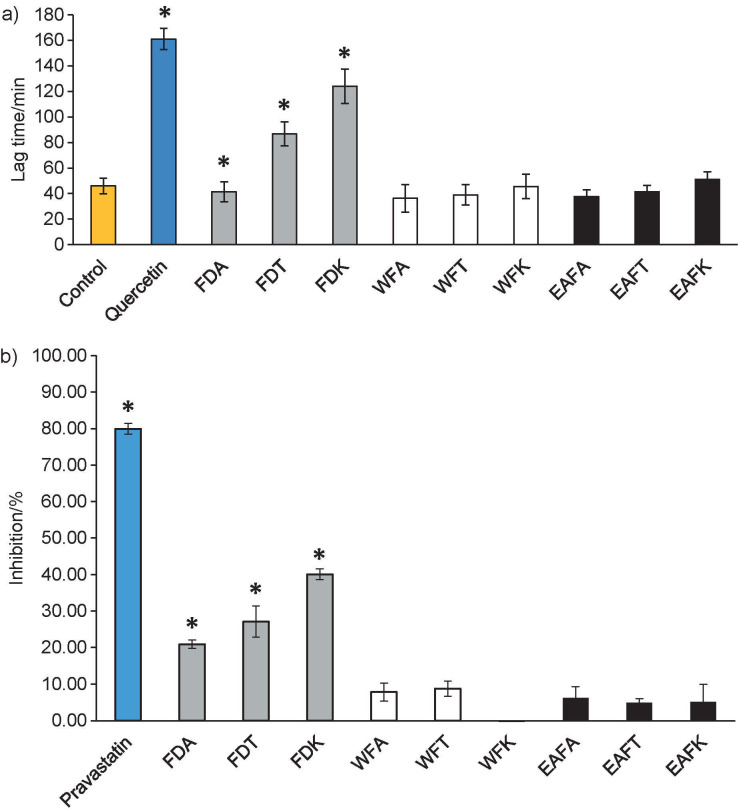
The inhibitory effect of: a) crude water extracts and fractions (*γ*=0.5 µg/mL) of different varieties of *Ficus deltoidea* var. *angustifolia* (FDA), *Ficus deltoidea* var. *trengganuensis* (FDT) and *Ficus deltoidea* var. *kunstleri* (FDK) on Fe^2+^-induced conjugated diene from low density lipoprotein (LDL). Values are displayed as mean±S.D. (*N*=4), and b) *F. deltoidea* (*γ*=5 µg/mL) on HMG-CoA reductase activity. The data were expressed as mean±S.D. (*N*=3). Negative control (orange bar), positive control (blue bar), crude extract (grey bar), water and ethyl acetate fraction of FDA, FDT and FDK (WFA, WFT and WFK, white bar and EAFA, EAFT and EAFK, black bar, respectively). Significance in comparison to the control is indicated by an asterisk (p<0.05)

The inhibitory actions of *Ficus deltoidea* varieties were assessed in this study using quantitative techniques including LDL oxidation and HMG-CoA reductase tests. Increased LDL level was previously reported as one of the risk factors associated with NAFLD (*19*). Oxidized LDL (oxLDL) can activate hepatic stellate cells, which play a key role in the pathogenesis of non-alcoholic steatohepatitis (NASH) (*20*). Previous studies have also reported higher levels of lipid peroxidation products such as oxLDL and thiobarbituric acid reactive substances (TBARS) in patients with NASH than their respective controls (*21*, *22*). FDK had the longest lag phase among the samples, indicating that it has a preventive effect against LDL oxidation (Fig. 1a). In addition, researchers have identified HMG-CoA reductase as a viable candidate for treating NAFLD (*23*). All crude water extracts of *F. deltoidea* leaves showed favourable inhibition activity towards HMG-CoA reductase, whereas minimal or no activity was observed in the water and ethyl acetate fractions (Fig. 1b). The inhibitory effect of crude extracts may have been brought on by a variety of phytochemicals that worked collectively. Even though *F. deltoidea* leaves exhibited a lower inhibitory activity than pravastatin, our findings may point to its potential mode of action, which involves preventing the production of endogenous cholesterol.

### Inhibition of lipid accumulation (steatosis) in palmitic acid-induced cells by F. deltoidea extracts

Natural remedies have been the focus of the hunt for a NAFLD cure in recent years because of their accessibility and degree of efficacy. Since palmitic acid, a saturated free fatty acid (FFA), is the most prevalent circulating FFA in the human body, particularly in plasma and tissues, as well as in the typical diet, it was utilised in our investigation to simulate steatotic conditions (*24*).

**Fig. 2:**
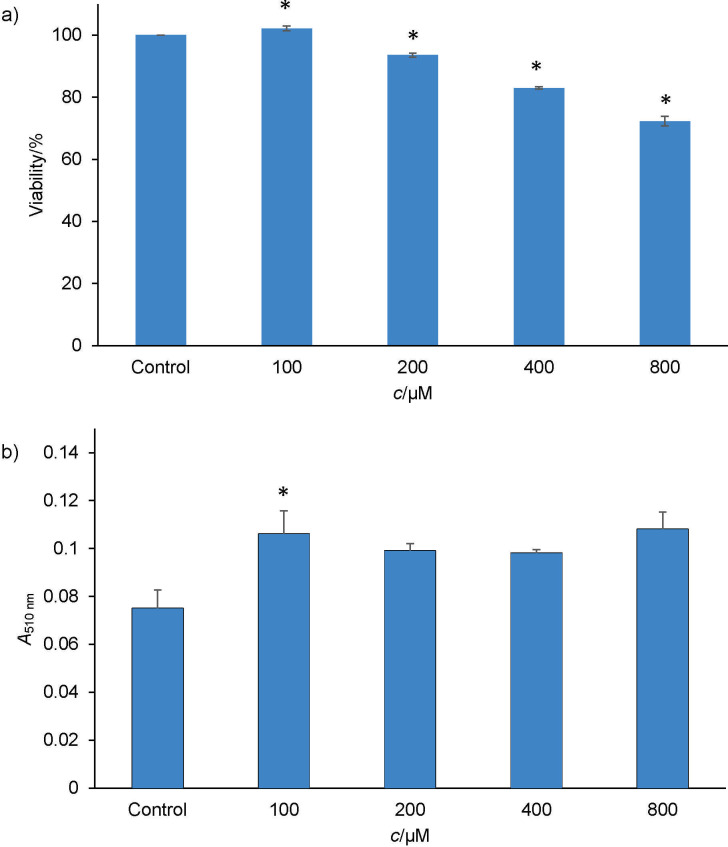
The results show: a) toxicity of palmitic acid (*c*=100–800 µM) on WRL68 cells after 24 h of incubation, and b) lipid accumulation in WRL68 cells after treatment with different concentrations of palmitic acid (*c*=100–800 µM). Values represent mean±S.D. (*N*=3). Significant differences from the control are denoted by asterisks (p<0.05)

**Fig. S2:**
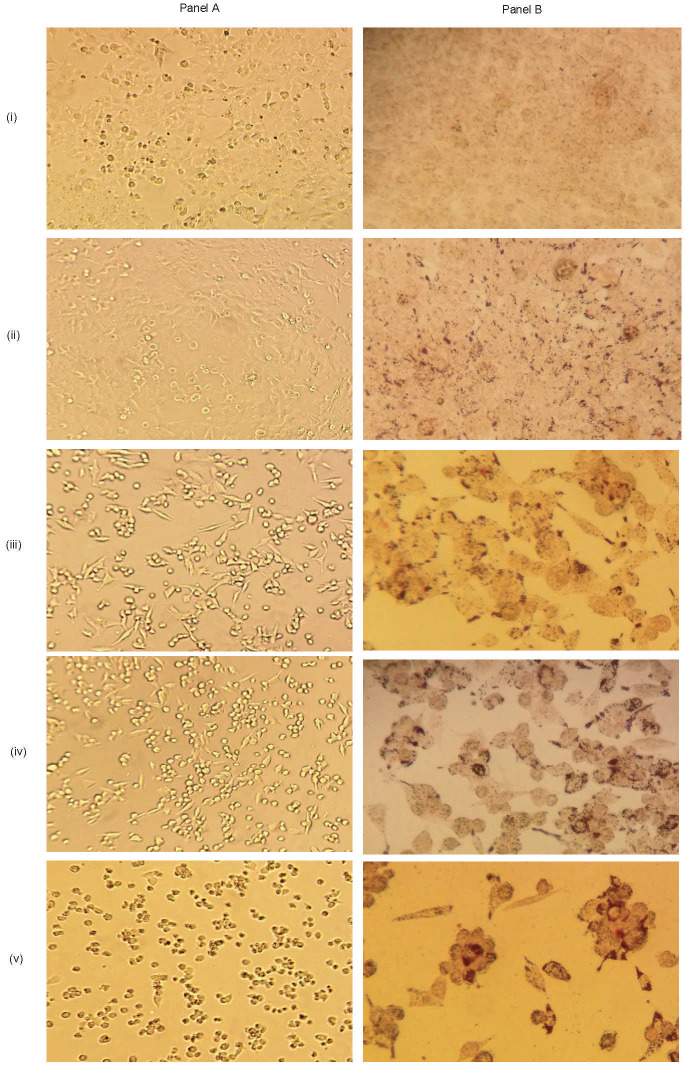
Images of the 3-(4,5-dimethylthiazol-2-yl)-2,5-diphenyl tetrazolium bromide (MTT) and Oil Red O assays of WRL68 cells treated with vehicle (bovine serum albumin) (i), and *c*(palmitic acid)=100 µM (ii), 200 µM (iii), 400 µM (iv), and 800 µM (v) are shown in panels A (10× magnification) and B (20× magnification), respectively

Based on Fig. 2a, percentage of cell viability decreased in a dose-dependent manner. The lowest concentration of palmitic acid (100 µM) shows no sign of toxicity, whereas at higher concentrations (200, 400 and 800 µM) a decrease in the cell viability was observed. Also, morphological changes of WRL68 cells treated with different concentrations of palmitic acid are shown in Fig. S2 (panel A). The same number of cells and concentrations of palmitic acid were tested on WRL68 cells to induce lipid accumulation and were observed using Oil Red O. Fig. 2b shows significant differences between the control and palmitic acid groups. The highest amount of lipid accumulation can be seen in the cells treated with 100 µM of palmitic acid compared to controls, while higher concentrations of palmitic acid (200, 400 and 800 µM) showed a reduction in lipid accumulation. Despite higher lipid accumulation, morphological changes such as shrinkage can be seen, as can empty spaces between the cells (Fig. S2, panel B). Thus, for this study, 100 µM of palmitic acid was chosen as the appropriate concentration to induce lipid accumulation and mimic the steatosis condition.

**Fig. 3:**
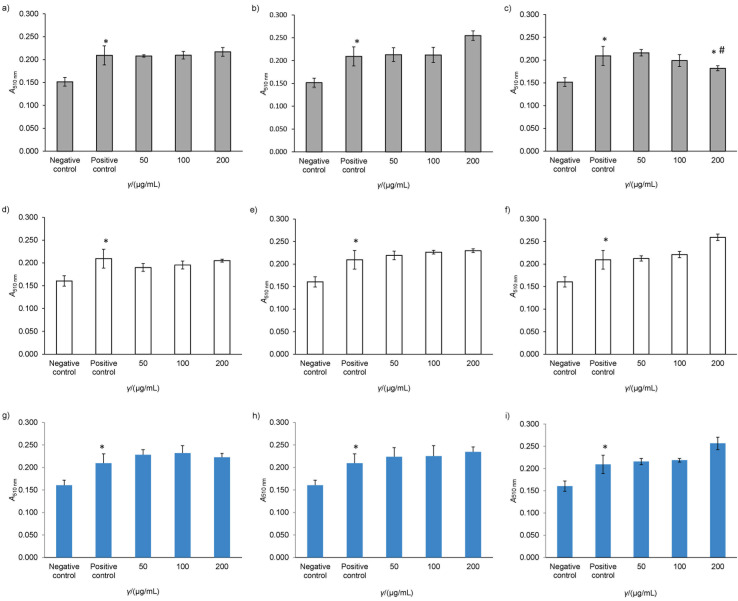
Lipid-reducing activity of *Ficus deltoidea* var. *angustifolia* (FDA; graphs a, d and g), *Ficus deltoidea* var. *trengganuensis* (FDT; graphs b, e and h) and *Ficus deltoidea* var. *kunstleri* (FDK; graphs c, f and i) in steatotic WRL68 cells measured with Oil Red O assay at different concentrations (*γ*=50–200 µg/mL). Crude extract (grey bar), water fraction (white bar), ethyl acetate fraction (blue bar). Values represent mean±S.D. (*N*=3). Symbols indicate the significance of the treatment group compared with the negative control (*) and positive control (#) groups (p<0.05). Negative control are cells incubated without palmitic acid or FDK, and positive control are cells incubated with palmitic acid

In Fig. 3, significant differences between the negative control and positive control groups were detected. Among all tested samples, only the crude extract of FDK showed a reduction in lipid accumulation. Treatment with FDK decreased significantly and in a dose-dependent manner the production of lipid droplets in WRL68 cells (Fig. 3c). Most of the samples, in contrast, lacked any inhibitory actions (Figs. 3a and 3b and Figs. 3d−3i). Plants and compounds of different species, such as fenugreek and rosmarinic acid, have been reported to possess the capacity to reduce lipid accumulation in different settings (*25*, *26*).

### FDK improves antioxidant status in steatotic palmitic acid-induced WRL68 cells

**Fig. 4:**
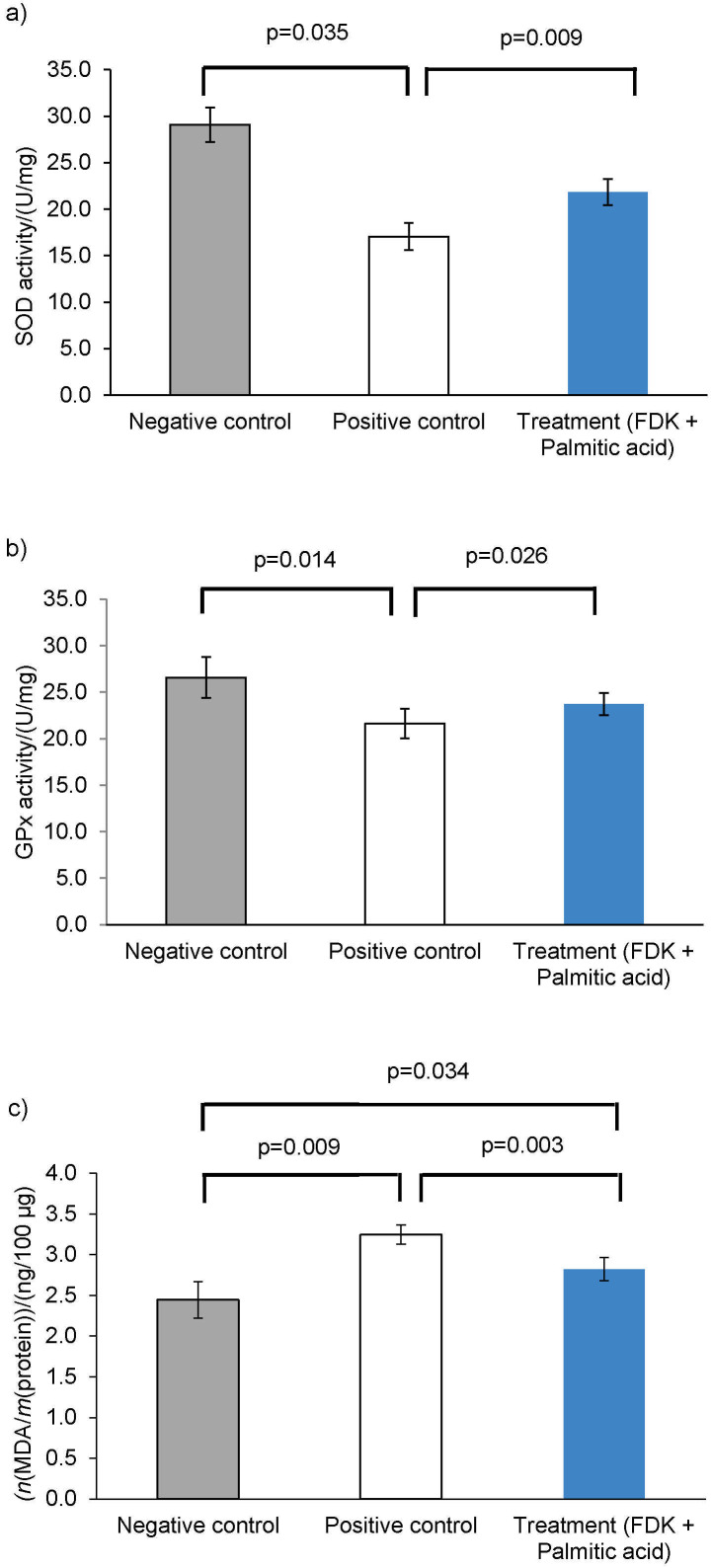
*Ficus deltoidea* var. *kunstleri* (FDK) crude extract lowers the imbalance of oxidative stress in steatotic WRL68 cells. The presence of *γ*(FDK)=200 µg/mL in palmitic acid-induced WRL68 cells had an impact on: a) superoxide dismutase (SOD), b) glutathione peroxidase enzymes (GPx), and c) malondialdehyde (MDA) production. Negative control (grey bar), positive control (white bar), treatment (blue bar). Values represent mean±S.D. (*N*=3). Negative control are cells incubated without the presence of palmitic acid or FDK and positive control are cells incubated with palmitic acid

According to studies, an excess of lipids in the liver can lead to inflammation, endoplasmic reticulum (ER) stress, mitochondrial malfunction, and a decrease in the ability of hepatocytes to produce endogenous antioxidants (*27*). Thus, NAFLD may be prevented and treated by lowering the accumulation of lipids and reducing oxidative stress. Some of the crucial oxidative stress indicators in NAFLD include SOD, GPx and MDA. Based on these results (Fig. 4a and Fig. 4b), cells incubated with palmitic acid caused a reduction of both SOD and GPx antioxidant enzymes in comparison to the control group, suggesting the cells were under oxidative stress. Even though the concentration of palmitic acid used in this study was not lethal, it did influence the oxidative status of the cells. The addition of FDK to the cells increased the activity of SOD and GPx, which demonstrates that FDK could aid in scavenging excess reactive oxygen species (ROS) produced by palmitic acid. Both enzymes play a critical role in first-line defences against cellular ROS accumulation.

Lipid peroxidation is another parameter of oxidative stress that has been assessed in this study. Steatosis, which occurs due to lipid accumulation, provides a potential substrate for lipid peroxidation and ROS toxicity. Excessive ROS accelerate the lipid peroxidation process, which then causes the liver to produce other reactive metabolites like MDA. Thus, inhibiting the lipid peroxidation is beneficial in the prevention and treatment of NAFLD. The addition of FDK in a pre-treatment step manages to reduce the formation of MDA in the treated cells (Fig. 4c). By preventing lipid peroxidation, this demonstrated the protective effect of FDK against palmitic acid lipotoxicity. Thus, based on the results, the increase of SOD and GPx activities, along with the reduction of MDA with FDK suggested an improvement of the oxidative balance in steatosis cells. Other studies have also shown that improving oxidative status in steatotic cells contributed to the lipid-reducing activities of plant extracts or compounds (*28*, *29*).

### Effect of FDK on protein expressions

**Fig. 5:**
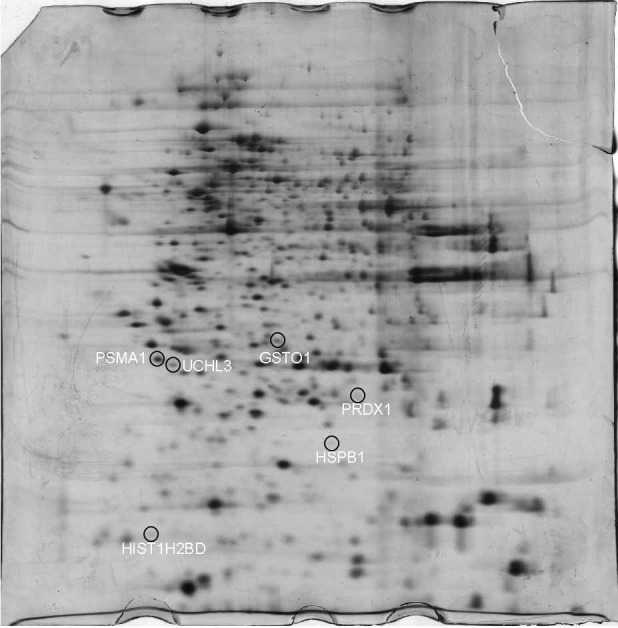
Proteome map of differentially expressed proteins in WRL68 cells. The protein spots were upregulated during incubation with *c*(palmitic acid)=100 µM and were downregulated when pretreated with the crude extract of *γ*(*Ficus deltoidea* var. *kunstleri*)=200 µg/mL (*N*=3, p≤0.05)

**Fig. S3:**
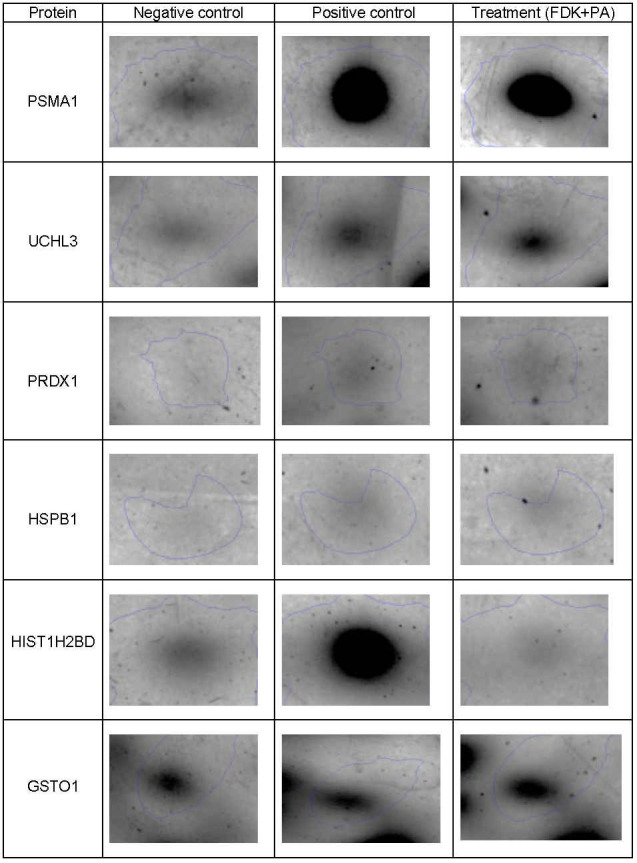
The differential expression in the negative control (cells incubated without the presence of palmitic acid or FDK), positive control (cells incubated with palmitic acid), and treatment (*γ*(FDK)=200 µg/mL+PA) in WRL68 cells was highlighted using enlarged pictures of the 2DE gel spots. FDK=*Ficus deltoidea* var. *kunstleri*, PA=palmitic acid

**Fig. S4:**
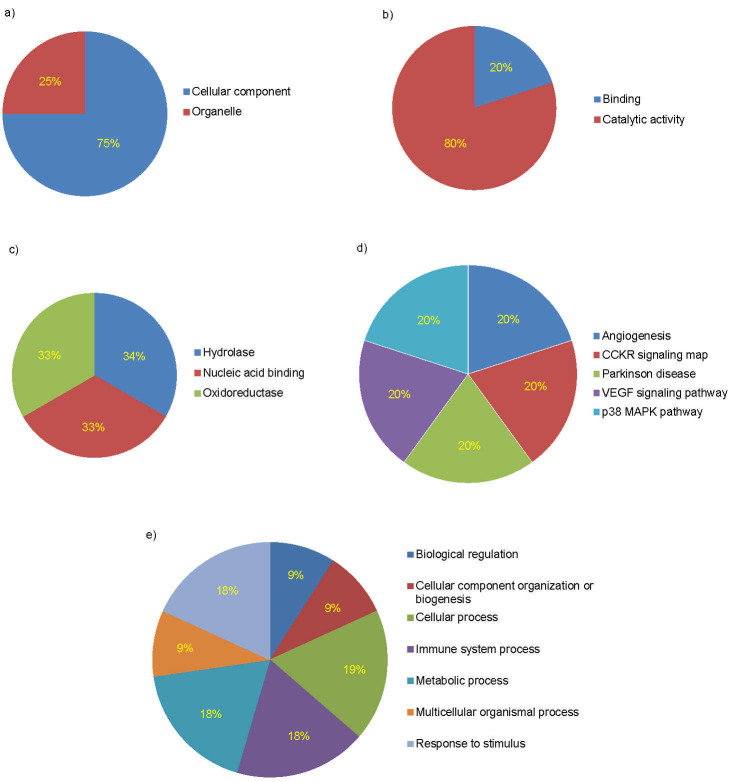
Pie charts representing the: a) cellular localization, b) molecular functions, c) protein classes, d) pathways, and e) biological processes of the identified proteins obtained from PANTHER database (*17*). The number of genes annotated to a particular category is inversely correlated with a sector's area

Using 2DE to identify changes in the protein abundance profile, the lipid-reducing effect of FDK was further studied (Fig. 5). Six proteins were discovered to be downregulated when pretreated with FDK and increased in the positive control group (Fig. S3). The protein spots HSPB1, UCHL3, GSTO1, PSMA1, HIST1H2BD and PRDX1 were successfully identified by MALDI-ToF/ToF (Table S1). According to the PANTHER database (*17*), most of the proteins that have been identified are cellular components that play a role in a variety of biological processes, including cellular operations, immune system functions, metabolic processes, and reactions to stimuli (Fig. S4). The proteins identified as being abundant in the Reactome pathway analysis are shown in Table S2. The proteins that have been discovered are linked to several pathways, primarily those that deal with protein homeostasis (UCH proteinases, protein ubiquitination, deubiquitination and neddylation), as well as the cellular stress response.

As previously shown, treatment with palmitic acid resulted in the downregulation of SOD and GPx and the increase of MDA in palmitic acid-induced WRL68 cells, which disrupted the balance of the cell redox regulation and had an impact on it, particularly in the endoplasmic reticulum (ER). The formation of unfolded/misfolded proteins, which lack specific functions in cellular metabolism, can occur because of any form of ER irregularity (*30*). The accumulation of misfolded proteins is toxic to the cells and results in the ER stress. Saturated fatty acids, such as palmitic acid, alter the phospholipid synthesis metabolic flux and increase the amount of saturated lipid species at the ER membrane, which may disrupt the fluidity of the membrane and encourage stiffening (*31*). The unfolded protein response (UPR), which is an adaptive reaction brought on by ER stress that restores protein homeostasis through intricate and parallel pathways, is brought on by an amplified state of these alterations. The activation of chaperone proteins is one of the UPR processes. This investigation discovered that palmitic acid increased the expression of HSPB1, a gene that is highly expressed in response to many environmental, physiological and pathological stressors (*32*). By subjecting oxidised proteins to the proteasome degradation machinery, this chaperone molecule avoids the aggregation of unfolded proteins and aids in protein breakdown (*33*).

Palmitic acid was also found to elevate the level of PSMA1, one of the essential protein subunits that contributes to the complex assembly of the 20S proteasome complex, involved in the ubiquitin-proteasome system (UPS). By destroying protein substrates that are no longer needed or are harmed by oxidative damage, the 20S proteasome is a molecular machine that plays a significant role in cellular activity (*34*). This proteasome is responsible for most of the non-lysosomal protein degradation in the cell, ensuring protein homeostasis (*35*). The upregulation of 20S proteasome is mediated by Nrf2 (nuclear factor erythroid 2-related factor 2), the master regulator of oxidative stress (*36*). The results in this study are in agreement with the findings by Soltis *et al.* (*37*), who found upregulation of PSMA1 in high fat diet-induced mice. Also, significantly altered PSMA1 level was reported and compared in the liver of healthy obese, normal control and steatosis samples (*38*).

UCHL3, a deubiquitinase related to UPS that removes ubiquitin from target proteins and controls their proteasomal breakdown and sub-localization, is another protein that was increased by palmitic acid. This maintains the equilibrium between ubiquitination and deubiquitination for the regulation of protein quality and homeostasis. The results of this investigation are consistent with a work by Suzuki *et al.* (*39*), who demonstrated that the reduction of UCHL3 induced an impairment in the adipogenesis that resulted in the prevention of lipid accumulation in adipocytes derived from UCHL3-/- mice. In contrast, van Beekum *et al.* (*40*) claimed that UCHL3 indirectly influenced peroxisome proliferator-activated receptor γ (PPAγ) activity by increasing β-oxidation.

Besides causing alterations in protein homeostasis, palmitic acid was found to cause changes in the levels of antioxidant enzymes. In this study, the level of PRDX1 was upregulated by palmitic acid, suggesting that it caused oxidative stress in the cells. However, pretreatment with FDK caused PRDX1 to decrease. The findings of van Greevenbroek *et al*. (*41*), who discovered that the level of PRDX1 expression in the liver of a hyperlipidaemic mouse model (HcB19) was twice as high as that of C3H mice, are supported by the current findings. Also, lipid accumulation in palmitate-induced insulin resistance HepG2 cells was reported to cause an up-regulation of PRDX1 expression (*42*). In contrast, PRDX1 was found to be reduced in MIN6 (mouse insulinoma) cells treated with palmitic acid (*43*). In response to H_2_O_2_, PRDX1 functions as a peroxide receptor, converting the signal into a disulfide bond that is then transmitted to ASK1 (apoptosis signalling kinase 1). This activation of the p38-MAPK (mitogen-activated protein kinase) pathway can affect various biological processes including inflammation, apoptosis, cell cycle and cell differentiation (*44*, *45*).

Another antioxidant enzyme that was affected by the palmitic acid treatment was GSTO1, which showed an increase in its activity. Overexpression of GSTO1 has been associated with increased protein glutathionylation (PG) that occurs during oxidative stress (*46*). By conjugating glutathione to the cysteine thiol group on the target proteins, PG is a redox-mediated post-translational modification that controls the activity of target proteins. Multiple biological processes are regulated by this mechanism, including those that control protein folding, cell signalling, inflammation and calcium homeostasis (*47*, *48*). PG is considered a defence mechanism to protect proteins from oxidative stress, a process that leads to irreversible damage (*49*). Also, an increase in glutathionylated proteins has been reported as part of NAFLD progression (*50*). In addition to PG, patients with NAFLD also had higher levels of protein carbonyls and 3-nitrotyrosine in their liver biopsies (*51*).

There have only been two studies on the ability of the 2DE approach to determine the decrease of lipid amount. Epigallocatechin gallate (EGCG) was investigated by Liu *et al*. (*52*) for its ability to reduce lipids in FFA-induced HepG2. The expression of proteins involved in the cytoskeleton, glycometabolism, signal transduction, DNA repair, mRNA processing, chaperone proteins, iron storage, protein phosphorylation, lipid metabolism, and antioxidant defence was found to be increased by FFA lipid build-up, but they were downregulated when EGCG was added to steatotic cells. The expression of the succinate dehydrogenase ubiquinone flavoprotein component, which is connected to cellular respiration, is downregulated by fat accumulation but upregulated by EGCG administration. In a different study, a methanol extract of *Tamarindus indica* was found to change the regulation of proteins in palmitic acid-induced HepG2 cells, including those involved in oxidative phosphorylation, metabolism, protein biosynthesis, cell proliferation and differentiation, and mRNA splicing (*53*). This change was reported to be the mechanism by which the extract lowers blood lipid levels. The number of significantly changed proteins was found to be lower than in the studies, according to the findings of this study. Different compounds, including fatty acids such as oleic acid, palmitic acid, *etc.*, concentration, duration, treatment and cell types employed to produce steatosis, could contribute to the difference (*54*). It is well known that diverse signalling pathways are involved in the cellular metabolic programme and are altered in varying degrees in NAFLD. Thus, many plants and compounds that have been tested produced variable results (*55*).

**Fig. S5:**
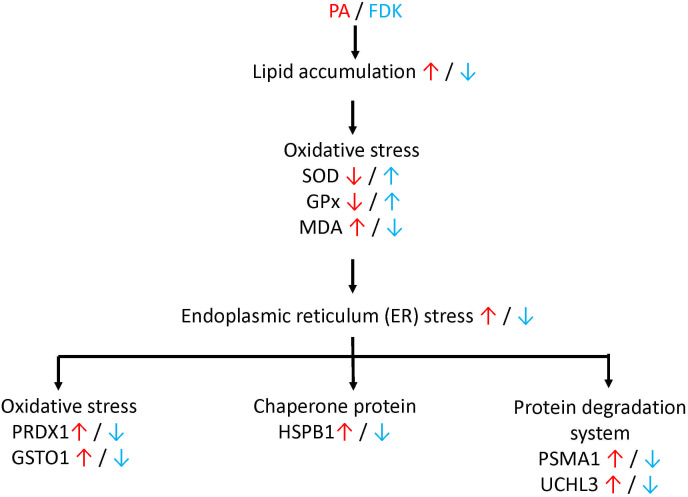
An overview of *Ficus deltoidea* var. *kunstleris* (FDK) effects in palmitic acid-induced WRL68 cells. The results of pretreatment with a crude water extract of FDK are shown in blue, while those caused by PA are shown in red. PA=palmitic acid, SOD=superoxide dismutase, GPx=glutathione peroxidase, MDA=malondialdehyde

According to our findings, lipid build-up in palmitic acid-induced WRL68 cells leads to oxidative stress, disturbs protein homeostasis and triggers the protein breakdown mechanism. We observed a decrease in lipid build-up after pre-treating these cells with FDK, which in turn helped to restore redox status and protein homeostasis (Fig. S5). In general, these findings may point to a protective mechanism of FDK against chronic liver disease, particularly in the early stages of NAFLD.

## CONCLUSIONS

In this research, palmitic acid (100 µM) was used to imitate steatosis in WRL68 cells, and the only sample that demonstrated a decrease in lipid accumulation was the crude water extract of *Ficus deltoidea* var. *kunstleri* (FDK). Superoxide dismutase (SOD), glutathione peroxidase (GPx) and malondialdehyde (MDA) - markers of oxidative stress - were used to further evaluate the impact of FDK. In palmitic acid-induced WRL68 cells, the aqueous extract of FDK (200 µg/mL) increased the SOD and GPx activities and decreased MDA, demonstrating its antioxidant properties. Palmitic acid was discovered to produce an increase of proteins associated with oxidative stress (PRDX1 and GSTO1) as well as protein homeostasis (HSPB1, PSMA1 and UCHL3) utilising a gel-based proteomics technique. A pre-treatment with FDK resulted in the downregulation of proteins, suggesting that FDK has a preventative function in NAFLD. Future research into the ability of *F. deltoidea* to reduce lipid content with a higher dose of palmitic acid to cause chronic steatosis would be intriguing.

## Appendix

**Table S2:** Alteration of protein abundance by the crude extract of *Ficus deltoidea* var. *kunstleri* (FDK) is associated with multiple pathways in steatotic WRL68 cells. The list shows the most significant pathways that involve the identified proteins sorted according to p-value (p<0.05). This data was obtained through the Reactome database (*18*)

	Pathway name	Found entity*	Identified protein	p-value	FDR
1	ABC-family protein mediated transport	2/161	HSPB1, PSMA1	0.005	0.143
2	UCH proteinases	2/172	PSMA1, UCHL3	0.005	0.143
3	G2/M checkpoints	2/194	PSMA1, HIST1H2BD	0.006	0.143
4	AUF1 (hnRNP D0) binds and destabilizes mRNA	3/198	HSPB1, PSMA1, GSTO1	0.007	0.143
5	RUNX1 regulates transcription of genes involved in differentiation of HSCs	2/230	PSMA1, HIST1H2BD	0.009	0.143
6	Apoptosis induced DNA fragmentation	1/17	HSPB1	0.011	0.143
7	Protein ubiquitination	2/275	UCHL3, HIST1H2BD	0.013	0.143
8	Deubiquitination	4/808	PSMA1, UCHL3, HIST1H2BD, PRDX1	0.013	0.143
9	Neddylation	2/292	PSMA1, UCHL3	0.014	0.143
10	Cellular response to stress	4/1650	PSMA1, HSPB1, PRDX1, HIST1H2BD	0.016	0.143

*The numbers of identified proteins from this study over total proteins that are related to that pathway. FDR=false discovery rate

**Table S1:** Crude extract of *Ficus deltoidea* var. *kunstleri* (FDK) alters protein abundance in steatotic WRL68 cells. Six significantly altered proteins were identified by MALDI-ToF/ToF in negative control (grey bar), positive control (white bar) and treatment (black bar) in steatotic WRL68 cells

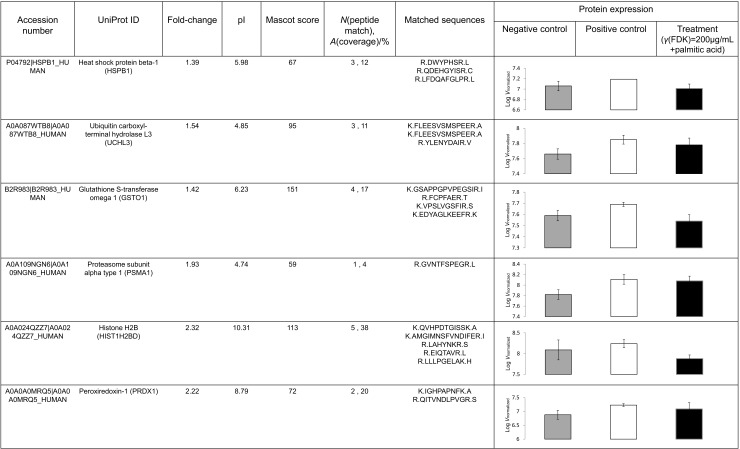

Negative control=cells incubated without the presence of palmitic acid or FDK, positive control=cells incubated with palmitic acid

## References

[1] Byrne CD Targher G. NAFLD: A multisystem disease. J Hepatol. 2015;62(1):S47–64. 10.1016/j.jhep.2014.12.012 25920090

[2] Jahn D, Kircher S, Hermanns HM, Geier A. Animal models of NAFLD from a hepatologist's point of view. BBA - Mol Basis Dis. 2019;1865(5):943-53. 10.1016/j.bbadis.2018.06.023 10.1016/j.bbadis.2018.06.023 29990551

[3] Ewang-Emukowhate M. Lipid-lowering agents. J Cardiovasc Pharmacol Ther. 2013;18(5):401–11. 10.1177 %2F1074248413492906 10.1177/1074248413492906 23811423

[4] Barter PJ Rye KA. New era of lipid-lowering drugs. Pharmacol Rev. 2016;68:458–75. 10.1124/pr.115.012203 26983688 PMC4813424

[5] Wilkinson MJ Laffin LJ Davidson MH. Overcoming toxicity and side-effects of lipid-lowering therapies. Best Pract Res Clin Endocrinol Metab. 2014;28(3):439–52. 10.1016/j.beem.2014.01.006 24840269

[6] Björnsson ES. Hepatotoxicity of statins and other lipid lowering agents. Liver Int. 2017;37:173–8. 10.1111/liv.13308 27860156

[7] Abu Bakar AR Manaharan T Merican AF Mohamad S. Experimental and computational approaches to reveal the potential of *Ficus deltoidea* leaves extract as α-amylase inhibitor. Nat Prod Res. 2018;32(4):473–6. 10.1080/14786419.2017.1312393 28391727

[8] Mohammad Noor HS Ismail NH Kasim N Mediani A Zohdi RM Ali AM Urinary metabolomics and biochemical analysis of antihyperglycemic effect of *Ficus deltoidea* Jack varieties in streptozotocin-nicotinamide–induced diabetic rats. Appl Biochem Biotechnol. 2020;192:1–21. 10.1007/s12010-020-03304-y 32215848

[9] Tantowi NCA Mohamed S Hussin P. Effect of *Ficus deltoidea*, a medicinal plant, on cartilage protection in cartilage explant and postmenopausal rat models of osteoarthritis. Osteoarthritis Cartilage. 2016;24 Suppl. 1:S353–4. 10.1016/j.joca.2016.01.636

[10] Nurdiana S Goh YM Hafandi A Dom SM Nur Syimal’ain A Noor Syaffinaz NM Improvement of spatial learning and memory, cortical gyrtification patterns and brain oxidative stress markers in diabetic rats treated with *Ficus deltoidea* leaf extract and vitexin. J Tradit Complement Med. 2018;8(1):190–202. 10.1016/j.jtcme.2017.05.006 29322009 PMC5755998

[11] Abrahim NN Abdul-Rahman PS Aminudin N. The antioxidant activities, cytotoxic properties, and identification of water-soluble compounds of *Ficus deltoidea* leaves. PeerJ. 2018;6:e5694. 10.7717/peerj.5694 30324012 PMC6186405

[12] Thuong PT Pokharel YR Lee MY Kim SK Bae K Su ND Dual anti-oxidative effects of fraxetin isolated from *Fraxinus rhinchophylla.* Biol Pharm Bull. 2009;32(9):1527–32. 10.1248/bpb.32.1527 19721227

[13] Razali N Aziz AA Lim CY Junit SM. Investigation into the effects of antioxidant-rich extract of *Tamarindus indica* leaf on antioxidant enzyme activities, oxidative stress and gene expression profiles in HepG2 cells. PeerJ. 2015;3:e1292. 10.7717/peerj.1292 26557426 PMC4636403

[14] Thio CLP Yusof R Ashrafzadeh A Bahari S Abdul-Rahman PS Karsani SA. Differential analysis of the secretome of WRL68 cells infected with the Chikugunya virus. PLoS One. 2015;10(6):e0129033. 10.1371/journal.pone.0129033 26083627 PMC4470940

[15] Rahim MAA Rahim ZHA Ahmad WAW Bakri MM Ismail MD Hashim OH. Inverse changes in plasma tetranectin and titin levels in patients with type 2 diabetes mellitus: A potential predictor of acute myocardial infarction? Acta Pharmacol Sin. 2018;39(7):1197–207. 10.1038/aps.2017.141 29417940 PMC6289394

[16] Boeckmann B Bairoch A Apweiler R Blatter MC Estreicher A Gasteiger E The SWISS-PROT protein knowledgebase and its supplement TrEMBL in 2003. Nucleic Acids Res. 2003;31(1):365–70. 10.1093/nar/gkg095 12520024 PMC165542

[17] Mi H Muruganujan A Casagrande JT Thomas PD. Large-scale gene function analysis with the PANTHER classification system. Nat Protoc. 2013;8(8):1551–66. 10.1038/nprot.2013.092 23868073 PMC6519453

[18] Joshi-Tope G Gillespie M Vastrik I D’Eustachio P Schmidt E de Bono B Reactome: A knowledgebase of biological pathways. Nucleic Acids Res. 2005;33 suppl_1:D428–32. 10.1093/nar/gki072 15608231 PMC540026

[19] Sun DQ Liu WY Wu SJ Zhu GQ Braddock M Zhang DC Increased levels of low-density lipoprotein cholesterol within the normal range as a risk factor for nonalcoholic fatty liver disease. Oncotarget. 2016;7(5):5728–37. 10.18632/oncotarget.6799 26735337 PMC4868717

[20] Kang Q Chen A. Curcumin suppresses expression of low‐density lipoprotein (LDL) receptor, leading to the inhibition of LDL‐induced activation of hepatic stellate cells. Br J Pharmacol. 2009;157(8):1354–67. 10.1111/j.1476-5381.2009.00261.x 19594758 PMC2765310

[21] Chalasani N Deeg MA Crabb DW. Systemic levels of lipid peroxidation and its metabolic and dietary correlates in patients with nonalcoholic steatohepatitis. Am J Gastroenterol. 2004;99(8):1497–502. 10.1111/j.1572-0241.2004.30159.x 15307867

[22] Davoodi I Rahimi R Abdollahi M Farzaei F Farzaei MH Memariani Z Promising effect of extract on high-fat diet induced nonalcoholic fatty liver. J Tradit Complement Med. 2017;7(4):508–14. 10.1016/j.jtcme.2017.01.008 29034200 PMC5634758

[23] Sun C Huang F Liu X Xiao X Yang M Hu G miR-21 regulates triglyceride and cholesterol metabolism in non-alcoholic fatty liver disease by targeting HMGCR. Int J Mol Med. 2015;35(3):847–53. 10.3892/ijmm.2015.2076 25605429

[24] Luo Y Rana P Will Y. Cyclosporine A and palmitic acid synergistically induce cytotoxicity in HepG2 cells. Toxicol Appl Pharmacol. 2012;261:172–80. 10.1016/j.taap.2012.03.022 22521608

[25] Vijayakumar MV Pandey V Mishra GC Bhat MK. Hypolipidemic effect of fenugreek seeds is mediated through inhibition of fat accumulation and upregulation of LDL receptor. Obesity (Silver Spring). 2010;18:667–74. 10.1038/oby.2009.337 19851306

[26] Balachander GJ Subramanian S Ilango K. Rosmarinic acid attenuates hepatic steatosis by modulating ER stress and autophagy in oleic acid-induced HepG2 cells. RSC Advances. 2018;8(47):26656–63. 10.1039/C8RA02849D 35547559 PMC9087887

[27] Spahis S Delvin E Borys JM Levy E. Oxidative stress as a critical factor in nonalcoholic fatty liver disease pathogenesis. Antioxid Redox Signal. 2017;26(10):519–41. 10.1089/ars.2016.6776 27452109

[28] Vidyashankar S Varma RS Patki PS. Quercetin ameliorate insulin resistance and up-regulates cellular antioxidants during oleic acid induced hepatic steatosis in HepG2 cells. Toxicol In Vitro. 2013;27(2):945–53. 10.1016/j.tiv.2013.01.014 23348005

[29] Khalil M Khalifeh H Baldini F Salis A Damonte G Daher A Antisteatotic and antioxidant activities of *Thymbra spicata* L. extracts in hepatic and endothelial cells as *in vitro* models of non-alcoholic fatty liver disease. J Ethnopharmacol. 2019;239:111919. 10.1016/j.jep.2019.111919 31029756

[30] Chong WC Shastri MD Eri R. Endoplasmic reticulum stress and oxidative stress: A vicious nexus implicated in bowel disease pathophysiology. Int J Mol Sci. 2017;18(4):771. 10.3390/ijms18040771 28379196 PMC5412355

[31] Ho N Xu C Thibault G. From the unfolded protein response to metabolic diseases–lipids under the spotlight. J Cell Sci. 2018;131:jcs199307. 10.1242/jcs.199307 29439157

[32] Weeks SD Muranova LK Heirbaut M Beelen S Strelkov SV Gusev NB. Characterization of human small heat shock protein HSPB1 α-crystallin domain localized mutants associated with hereditary motor neuron diseases. Sci Rep. 2018;8(1):688. 10.1038/s41598-017-18874-x 29330367 PMC5766566

[33] Vendredy L Adriaenssens E Timmerman V. Small heat shock proteins in neurodegenerative diseases. Cell Stress Chaperones. 2020;25(4):679–99. 10.1007/s12192-020-01101-4 32323160 PMC7332613

[34] Raynes R Pomatto LC Davies KJ. Degradation of oxidized proteins by the proteasome: distinguishing between the 20S, 26S, and immunoproteasome proteolytic pathways. Mol Aspects Med. 2016;50:41–55. 10.1016/j.mam.2016.05.001 27155164 PMC4967006

[35] Latham MP Sekhar A Kay LE. Understanding the mechanism of proteasome 20S core particle gating. Proc Natl Acad Sci USA. 2014;111(15):5532–7. 10.1073/pnas.1322079111 24706783 PMC3992659

[36] Pickering AM, Linder RA, Zhang H, Forman HJ, Davies KJ. Nrf2-dependent induction of proteasome and Pa28αβ regulator are required for adaptation to oxidative stress. J Biol. 201;287(13):10021-31. 10.1074/jbc.M111.277145 10.1074/jbc.M111.277145 PMC3323025 22308036

[37] Soltis AR Kennedy NJ Xin X Zhou F Ficarro SB Yap YS Hepatic dysfunction caused by consumption of a high-fat diet. Cell Rep. 2017;21(11):3317–28. 10.1016/j.celrep.2017.11.059 29241556 PMC5734865

[38] Wang R Wang X Zhuang L. Gene expression profiling reveals key genes and pathways related to the development of non-alcoholic fatty liver disease. Ann Hepatol. 2016;15(2):190–9. 10.5604/16652681.1193709 26845596

[39] Suzuki M Setsuie R Wada K. Ubiquitin carboxyl-terminal hydrolase l3 promotes insulin signalling and adipogenesis. Endocrinology. 2009;150(12):5230–9. 10.1210/en.2009-0332 19837878

[40] van Beekum O Gao Y Berger R Koppen A Kalkhoven E. A novel RNAi lethality rescue screen to identify regulators of adipogenesis. PLoS One. 2012;7(6):e37680. 10.1371/journal.pone.0037680 22679485 PMC3367974

[41] van Greevenbroek MM Vermeulen VMM de Bruin TW. Identification of novel molecular candidates for fatty liver in the hyperlipidemic mouse model, HcB19. J Lipid Res. 2004;45(6):1148–54. 10.1194/jlr.M400062-JLR200 15060090

[42] Tang Z Xia N Yuan X Zhu X Xu G Cui S PRDX1 is involved in palmitate induced insulin resistance via regulating the activity of p38MAPK in HepG2 cells. Biochem Biophys Res Commun. 2015;465(4):670–7. 10.1016/j.bbrc.2015.08.008 26301632

[43] Sargsyan E Artemenko K Manukyan L Bergquist J Bergsten P. Oleate protects beta-cells from the toxic effect of palmitate by activating pro-survival pathways of the ER stress response. Biochim Biophys Acta. 2016;1861(9 Pt A):1151–60. 10.1016/j.bbalip.2016.06.012 27344025

[44] Zarubin T Jiahuai HAN. Activation and signaling of the p38 MAP kinase pathway. Cell Res. 2005;15:11–8. 10.1038/sj.cr.7290257 15686620

[45] Jarvis RM Hughes SM Ledgerwood EC. Peroxiredoxin 1 functions as a signal peroxidase to receive, transduce, and transmit peroxide signals in mammalian cells. Free Radic Biol Med. 2012;53(7):1522–30. 10.1016/j.freeradbiomed.2012.08.001 22902630

[46] Kim Y Cha SJ Choi HJ Kim K. Omega class glutathione s-transferase: antioxidant enzyme in pathogenesis of neurodegenerative diseases. Oxid Med Cell Longev. 2017;2017:5049532. 10.1155/2017/5049532 29435097 PMC5757135

[47] Armeni T Ercolani L Urbanelli L Magini A Magherini F Pugnaloni A Cellular redox imbalance and changes of protein S-glutathionylation patterns are associated with senescence induced by oncogenic H-ras. PLoS One. 2012;7(12):e52151. 10.1371/journal.pone.0052151 23284910 PMC3527427

[48] Cha SJ Kim H Choi HJ Lee S Kim K. Protein glutathionylation in the pathogenesis of neurodegenerative diseases. Oxid Med Cell Longev. 2017;2017:2818565. 10.1155/2017/2818565 29456785 PMC5804111

[49] Boguszewska-Mańkowska D, Nykiel M, Zagdańska B. Protein oxidation and redox regulation of proteolysis. In: Gowder SJT, editor. Basic principles and clinical significance of oxidative stress. London, UK: IntechOpen Ltd; 2015. pp. 17-35. 10.5772/61182 10.5772/61182

[50] Piemonte F Petrini S Gaeta LM Tozzi G Bertini E Devito R Protein glutathionylation increases in the liver of patients with non‐alcoholic fatty liver disease. J Gastroenterol Hepatol. 2008;23(8 Pt 2):e457–64. 10.1111/j.1440-1746.2007.05070.x 17683488

[51] Liu W Baker SS Baker RD Zhu L. Antioxidant mechanisms in nonalcoholic fatty liver disease. Curr Drug Targets. 2015;16(12):1301–14. 10.2174/1389450116666150427155342 25915484

[52] Liu Z Li Q Huang J Liang Q Yan Y Lin H Proteomic analysis of the inhibitory effect of epigallocatechin gallate on lipid accumulation in human HepG2 cells. Proteome Sci. 2013;11(1):32. 10.1186/1477-5956-11-32 23866759 PMC3723827

[53] Chong URW Abdul-Rahman PS Abdul-Aziz A Hashim OH Mat-Junit S. Effects of *Tamarindus indica* fruit pulp extract on abundance of HepG2 cell lysate proteins and their possible consequential impact on metabolism and inflammation. BioMed Res Int. 2013;2013:459017. 10.1155/2013/459017 24455694 PMC3886566

[54] Alsabeeh N Chausse B Kakimoto PA Kowaltowski AJ Shirihai O. Cell culture models of fatty acid overload: problems and solutions. Biochim Biophys Acta Mol Cell Biol Lipids. 2018;1863(2):143–51. 10.1016/j.bbalip.2017.11.006 29155055 PMC5969525

[55] Gusdon AM Song KX Qu S. Nonalcoholic fatty liver disease: pathogenesis and therapeutics from a mitochondria-centric perspective. Oxid Med Cell Longev. 2014;2014:637027. 10.1155/2014/637027 25371775 PMC4211163

